# *Porphyromonas gingivalis*: Major Periodontopathic Pathogen Overview

**DOI:** 10.1155/2014/476068

**Published:** 2014-03-25

**Authors:** Jaroslav Mysak, Stepan Podzimek, Pavla Sommerova, Yelena Lyuya-Mi, Jirina Bartova, Tatjana Janatova, Jarmila Prochazkova, Jana Duskova

**Affiliations:** Institute of Clinical and Experimental Dental Medicine, First Faculty of Medicine and General University Hospital, Charles University, Karlovo Namesti 32, 12000 Prague, Czech Republic

## Abstract

*Porphyromonas gingivalis* is a Gram-negative oral anaerobe that is involved in the pathogenesis of periodontitis and is a member of more than 500 bacterial species that live in the oral cavity. This anaerobic bacterium is a natural member of the oral microbiome, yet it can become highly destructive (termed pathobiont) and proliferate to high cell numbers in periodontal lesions: this is attributed to its arsenal of specialized virulence factors. The purpose of this review is to provide an overview of one of the main periodontal pathogens—*Porphyromonas gingivalis.* This bacterium, along with *Treponema denticola* and *Tannerella forsythia*, constitute the “red complex,” a prototype polybacterial pathogenic consortium in periodontitis. This review outlines *Porphyromonas gingivalis* structure, its metabolism, its ability to colonize the epithelial cells, and its influence upon the host immunity.

## 1. Introduction

*Porphyromonas gingivalis* is a Gram-negative oral anaerobe that is involved in the pathogenesis of periodontitis, an inflammatory disease that destroys the tissues supporting the tooth which eventually may lead to tooth loss. Among the over 500 bacterial species living in the oral cavity, a bacterial complex named “red complex” and composed of* Porphyromonas gingivalis*,* Treponema denticola*, and* Tannerella forsythia* has been strongly associated with advanced periodontal lesions [1].

Research on* Porphyromonas gingivalis*, a periodontopathogen, has provided a tremendous amount of information in terms of phylogenetic as well as proteomic criteria over the last 20 years, which may exceed other closely related members, including* Bacteroides fragilis* and* Bacteroides thetaiotaomicron*, as major anaerobic, opportunistic pathogens in the dental field. The microbiota of the human oral mucosa consists of a myriad of bacterial species that normally exist in commensal harmony with the host.* Porphyromonas gingivalis*, an etiological agent in severe forms of periodontitis (a chronic inflammatory disease), is a prominent component of the oral microbiome and a successful colonizer of the oral epithelium [2].

The authors have used both latest as well as older reviews and studies to compile a comprehensive overview of this key periodontal pathogen. Special attention has been paid to clinically relevant properties of* Porphyromonas gingivalis*, such as pathogenicity and its possible relation to certain systemic diseases.

## 2. *Porphyromonas gingivalis*

### 2.1. Properties and Pathogenicity of* Porphyromonas gingivalis*

The perturbation of epithelial cells by bacteria is the first stage in the initiation of inflammatory and immune processes which eventually cause destruction of the tissues surrounding and supporting the teeth which ultimately result in tooth loss [3].

*Porphyromonas gingivalis* can locally invade periodontal tissues and evade the host defense mechanisms. In doing so, it utilizes a panel of virulence factors that cause deregulation of the innate immune and inflammatory responses [4].

*Porphyromonas gingivalis* rapidly adheres to the host cell surface followed by internalization via lipid rafts and incorporation of the bacterium into early phagosomes.* Porphyromonas gingivalis* activates cellular autophagy to provide a replicative niche while suppressing apoptosis. The replicating vacuole contains host proteins delivered by autophagy that are used by this asaccharolytic pathogen to survive and replicate within the host cell. When autophagy is suppressed by 3-methyladenine or wortmannin, internalized* Porphyromonas gingivalis* transits to the phagolysosome where it is destroyed and degraded. For that reason, the survival of* Porphyromonas gingivalis* depends upon the activation of autophagy and survival of the endothelial host cell, but the mechanism by which* Porphyromonas gingivalis* accomplishes this remains unclear [5].

The harsh inflammatory condition of the periodontal pocket suggests that this organism has properties that will facilitate its ability to respond and adapt to oxidative stress. Because the stress response in the pathogen is a major determinant of its virulence, a comprehensive understanding of its oxidative stress resistance strategy is vital [6].

The ability of* Porphyromonas gingivalis* to cause adult periodontitis is determined by its arsenal of virulence factors. Biofilm formation and bacterial dipeptidyl peptidase IV (DPPIV) activity contribute to the pathogenic potential of* Porphyromonas gingivalis*. Furthermore, biofilm formation may enhance* Porphyromonas gingivalis* virulence through increased DPPIV activity. Because of their importance for bacterial colonization and growth, biofilm formation and DPPIV activity could present interesting therapeutic targets to tackle periodontitis [7].

*Porphyromonas gingivalis* is strongly correlated with chronic periodontitis. Its chronic persistence in the periodontium depends on its ability to evade host immunity without inhibiting the overall inflammatory response, which is actually beneficial for this and other periodontal bacteria. Indeed, the inflammatory exudate (gingival crevicular fluid) is a source of essential nutrients, such as peptides and hemin-derived iron [8].

*Porphyromonas gingivalis* contributes to the pathogenesis of aggressive periodontitis by inducing high levels of proinflammatory cytokines, such as IL-1*β* and IL-6 by peripheral CD4^+^ T helper cells [9].* Porphyromonas gingivalis* serotypes K1 and K2 but not others are associated with an increased production of the osteoclastogenesis-related factor RANKL. This important information suggests that these serotypes could elicit a greater bone resorption in vivo and have a significant role in the periodontitis pathogenesis. Destructive periodontitis is associated with a Th1-Th17-immune response and activation of RANKL-induced osteoclasts. In addition,* Porphyromonas gingivalis* K1 and K2 serotypes induce a strong Th1-Th17-response. These* Porphyromonas gingivalis* serotypes induce higher osteoclasts activation, by increased Th17-associated RANKL production and an antigen-specific memory T lymphocyte response [10]. Chronic* Porphyromonas gingivalis* oral infection prior to arthritis induction increases the immune system activation favoring Th17 cell responses which ultimately accelerate arthritis development. These results suggest that chronic oral infection may influence rheumatoid arthritis development mainly through activation of Th17-related pathways [11].

Salivary concentrations of metalloproteinase MMP-8, interleukin IL-1*β*, and* Porphyromonas gingivalis* are associated with various clinical and radiographic measures of periodontitis. The CRS index, combining the three salivary biomarkers, is associated with periodontitis. High salivary concentrations of metalloproteinase MMP-8, interleukin IL-1*β*, and* Porphyromonas gingivalis* have been associated with deepened periodontal pockets and alveolar bone loss and MMP-8 and IL-1*β* with bleeding on probing [12].

The bacterium utilizes amino acids as energy and carbon sources and incorporates them mainly as dipeptides. Therefore, a wide variety of dipeptide production processes mediated by dipeptidyl peptidases (DPPs) could be beneficial for the organism [13].

### 2.2. Virulence and Growth of* Porphyromonas gingivalis*: Role of Iron

Iron utilized by this pathogen in the form of heme has been shown to play an essential role in its growth and virulence.* Porphyromonas gingivalis* does not produce siderophores. Instead, it employs specific outer membrane receptors, proteases (particularly gingipains), and lipoproteins to acquire iron/heme. Specific proteins involved in iron and heme capture have been described [14].

Additionally, the proteolytic activities of gingipain R and gingipain K contribute to processing/maturation of various cell-surface proteins of* Porphyromonas gingivalis*, such as fimA fimbrilin (a subunit of major fimbriae), 75-kDa protein (a subunit of minor fimbriae), hemagglutinins, and the hemoglobin receptor protein, which are important for the bacterium to colonize and proliferate in the gingival crevice and to invade the periodontium. These findings strongly point out the critical roles of gingipain R and gingipain K in the virulence of* Porphyromonas gingivalis* [15]. Protease gingipain R exists as 110-, 95-, and 70- to 90- and 50-kDa proteins, the first two being a complex of the 50-kDa catalytic subunit with hemagglutinin/adhesins, with or without an added membrane anchorage peptide. The other forms are single-chain enzymes. The predominant form of gingipain K in* Porphyromonas gingivalis* strains is a complex of a 60-kDa catalytic protein with hemagglutinin/adhesins. Molecular cloning and structural characterization of the gingipain R and gingipain K genes has shown that they code for 1704 and 1722 amino-acid residue preproenzymes, respectively [16]. The virulence of argingipain was further substantiated by disruption of argingipain-encoding genes on the chromosome by use of suicide plasmid systems. On the other hand, the roles of host cell-derived proteinases in the periodontal tissue breakdown have been studied [17]. Levels of lysosomal proteinases such as cathepsins B, H, L, and G and medullasin were determined in gingival crevicular fluid from periodontitis patients and experimental gingivitis subjects by activity measurement and sensitive immunoassay [17].

### 2.3. Virulence and Growth of* Porphyromonas gingivalis*: Role of Adhesins

Retention and growth of* Porphyromonas gingivalis*on diverse surfaces are facilitated by a repertoire of adhesins including fimbriae, hemagglutinins, and proteinases [18]. Histatins are human salivary gland peptides with antimicrobial and anti-inflammatory activities. We hypothesized that histatin 5 binds to* Porphyromonas gingivalis* hemagglutinin B (HagB) and attenuates HagB-induced chemokine responses in human myeloid dendritic cells. Thus histatin 5 is capable of attenuating chemokine responses which may help control oral inflammation [19].

*Porphyromonas gingivalis* produces large amounts of arginine- and lysine-specific cysteine proteinases in cell-associated and secretory forms [20].

These enzymes, referred to as gingipains, cleave protein and peptide substrates after arginine (gingipain R) and lysine residues (gingipain K), and it has been found [21] that neither is easily inhibited by host proteinase inhibitors. Examination of the properties of each proteinase clearly indicates a role(s) for both in the dysregulation of a number of normally tightly controlled pathways. The effects of such uncontrolled proteolysis are the development of edema (kallikrein/kinin pathway activation by gingipain R), neutrophil infiltration (complement pathway activation by gingipain R), and bleeding (degradation of fibrinogen by gingipain K) [21]. The crystal structure of the K1 domain, an adhesin module of the lysine gingipain (Kgp) expressed on the cell surface by the periodontopathic anaerobic bacterium,* Porphyromonas gingivalis* W83, is comparable to the previously determined structures of homologues K2 and K3, all three being representative members of the cleaved adhesin domain family. In the structure of K1, the conformation of the most extensive surface loop is unexpectedly perturbed, perhaps by crystal packing, and is displaced from the previously reported arginine-anchored position observed in K2 and K3. This displacement allows the loop to become free to interact with other proteins; the alternate flipped-out loop conformation is a novel mechanism for interacting with target host proteins, other bacteria, or other gingipain protein domains. Furthermore, the K1 adhesin module, like others, is found to be hemolytic in vitro and thus functions in erythrocyte recognition contributing to the hemolytic function of Kgp. K1 was also observed to selectively bind to hemalbumin with high affinity, suggesting this domain may be involved in gingipain-mediated hem acquisition from hemalbumin. Consequently, it is most likely that all cleaved adhesin domains of Kgp contribute to the pathogenicity of* Porphyromonas gingivalis* in more complex ways than simply mediating bacterial adherence [22].

The presence of antibodies to the* Porphyromonas gingivalis*-specific virulence factor arginine gingipainB was screened by ELISA method. Significantly higher anti-RgpB antibody levels were detected in the periodontitis subset, compared to the nonperiodontitis control subset. Significantly increased anti-RgpB antibody levels in periodontitis serum, compared to nonperiodontitis serum, may suggest that elevated anti-RgpB IgG levels can be used as a proxy for chronic periodontitis in studies where the periodontal status is of interest but unknown [23].

*Porphyromonas gingivalis* HmuY hemophore-like protein binds heme and scavenges heme from host hemoproteins to further deliver it to the cognate heme receptor HmuR. The characterization of structural features of HmuY variants in the presence and absence of heme with respect to roles of tryptophan residues in conformational stability is the aim of the further research [24].

*Porphyromonas gingivalis* contains exceedingly high concentrations of cysteine proteinases with trypsin-like activity which has been implicated as a virulence factor in adult-onset periodontitis [21]. Several of these enzymes are apparently expressed as active proteolytic products following processing of larger precursor proteins. In addition, more recent data [25] have suggested a close relationship between some of these enzymes and two other potential virulence factors of* Porphyromonas gingivalis*: hemagglutinins and collagenases [25].

Surface components of* Porphyromonas gingivalis* have contact with host tissues and cells because of the outermost cell elements. As a result, such components of* Porphyromonas gingivalis* are potentially important in the occurrence of periodontal diseases [26].* Porphyromonas gingivalis* fimbriae are a critical factor for mediation of interaction of this bacterial organism with host tissues, as* Porphyromonas gingivalis* promotes both bacterial adhesion to and invasion of targeted sites.* Porphyromonas gingivalis* fimbriae are likely to interrupt the cellular signaling via extracellular matrix proteins/integrins in periodontal regions. Fimbriae are also thought to be critically important in invasive events of this bacterial organism to host cells [27].

*Porphyromonas gingivalis* fimbriae are capable of binding to salivary enzymes, extracellular matrix proteins, and commensal bacteria as well as also strongly adhering to cellular alpha5beta1-integrin [28]. Following adhesion to alpha5beta1-integrin,* Porphyromonas gingivalis* is captured by cellular pseudopodia which enable invagination through an actin-mediated pathway. The invasive event has been reported to require host cellular dynamin, actin fibers, microtubules, and lipid rafts [28]. Following passage through the epithelial barrier, the intracellular* Porphyromonas gingivalis* pathogen impairs cellular function.* Porphyromonas gingivalis* fimbriae are classified into 6 genotypes (types I to V and Ib) based on the diversity of the fimA genes encoding each fimbria subunit. Intracellular* Porphyromonas gingivalis* with type II fimbriae has been found to clearly degrade integrin-related signaling molecules, paxillin and focal adhesion kinase which disables cellular migration and proliferation of the host cells [28]. A majority of periodontitis patients were found to carry type II fimA fibriae of* Porphyromonas gingivalis*, followed by type IV; type II fimA fimbriae of* Porphyromonas gingivalis* were found significantly more often in patients with more severe forms of periodontitis [29].* Porphyromonas gingivalis* fimbriae act as an important virulence factor in atherosclerosis progression. Regulatory T cells (Tregs) may play a crucial role in autoimmune response during this process. However, whether* Porphyromonas gingivalis* infection is associated with Tregs dysregulation during atherosclerosis is still unknown and the prevalence of different* Porphyromonas gingivalis* FimA genotypes during this process is unclear.* Porphyromonas gingivalis* infection reduced Tregs in atherosclerotic patients [30].

### 2.4. Lipopolysaccharide of* Porphyromonas gingivalis*

The lipopolysaccharide of* Porphyromonas gingivalis* is a key factor in the development of periodontitis. Gingival fibroblasts, which are the major constituents of gingival connective tissue, may directly interact with* Porphyromonas gingivalis* and its bacterial products, including lipopolysaccharide, in periodontitis lesions [31]. Due to its ability to potently activate host inflammatory and innate defense responses, it has been proposed to function as an important molecule that alerts the host of potential bacterial infection. However, although highly conserved, lipopolysaccharide contains important structural differences among different bacterial species that can significantly alter host responses [32]. Plasminogen activator inhibitor type 1 (PAI-1) mRNA binding protein expression was increased in gingiva from periodontitis patients [33].

*Porphyromonas gingivalis* lipopolysaccharide increases the expression of EphB4 while inhibiting the expression of EphrinB2 [34].* Porphyromonas gingivalis* LPS induces proinflammatory cytokines, such as IL-1*β*, IL-6, and IL-8, which induce periodontal tissue destruction. Periodontal ligament stem cells (PDLSCs) play an important role in periodontal tissue regeneration and are expected to have future applications in cellular therapies for periodontitis.* Porphyromonas gingivalis* LPS inhibited the alkaline phosphatase activity, collagen type 1 Alpha 1, and osteocalcin production and mineralization in the PDLSCs which are positive for STRO-1 and SSEA-4.* Porphyromonas gingivalis* LPS also promoted cell proliferation and produced IL-1*β*, IL-6, and IL-8 [35].

The effect of* Porphyromonas gingivalis* LPS was compared with that of Toll-like receptor 2 agonist synthetic triacylated lipoprotein Pam3-Cys-Ser-(Lys)4 (Pam3CSK4). Gene and protein expression of intercellular adhesion molecule-1, vascular cell adhesion molecule-1, and E-selectin could be measured using RT-PCR and flow cytometry, respectively.* Porphyromonas gingivalis* LPS stimulates the expression levels of all adhesion molecules in a dose-dependent manner [36].

### 2.5. Cysteine Proteases of* Porphyromonas gingivalis*

The cysteine proteases of* Porphyromonas gingivalis* are extracellular products of an important etiological agent in periodontal diseases. Many of the in vitro actions of these enzymes are consistent with the observed deregulated inflammatory and immune features of the disease [37]. They are significant targets of the immune responses of affected individuals and are viewed by some as potential molecular targets for therapeutic approaches to periodontal diseases [37]. These enzymes are involved in both the destruction of periodontal tissues and interrupting host-defense mechanisms through the degradation of immunoglobulins and complement factors leading eventually to disease progression [38]. The utilization of monospecific mutants defective in individual proteases has demonstrated that protease activity is important in virulence but also has suggested the complexity of the functions of the enzymes in the physiology of these microorganisms [39].

### 2.6. *Porphyromonas gingivalis* and Complement System

The complement system is an important host response to invading bacteria. Activation of the complement leads to deposition of C3b on the bacterial surface and phagocytosis of the opsonized bacteria by host cells. Alternatively, the entire complement pathway, including terminal components C5b-9, may be activated on the cell surface which gives rise to generation and insertion of the membrane attack complex into the bacterial membrane and cell lysis. Bacterial resistance to complement may be by enzyme digestion of complement components or by the generation or acquisition from the host of cell surface molecules which allow the organism to adopt host complement control proteins [40]. The proadhesive pathway is initiated when* Porphyromonas gingivalis* fimbriae bind CD14 and activate Toll-like receptor 2 (TLR2) and phosphatidylinositol 3-kinase-mediated signaling leading to induction of the high-affinity conformation of CR3 in leukocytes. Although this TLR2 proadhesive signaling pathway may normally be involved in enhancing leukocyte-endothelial cell interactions and transendothelial migration, intriguing evidence [41] suggests that* Porphyromonas gingivalis* has co-opted this pathway for enhancing the interaction of its cell surface fimbriae with CR3. Indeed, activated CR3 interacts with* Porphyromonas gingivalis* fimbriae and induces downregulation of interleukin-12 p70, a key cytokine involved in intracellular bacterial clearance. Moreover, the interaction of activated CR3 with* Porphyromonas gingivalis* leads to the internalization of the pathogen by macrophages. Since CR3 does not readily activate microbicidal mechanisms and constitutes a “preferred receptor” for certain intracellular pathogens, possible exploitation of CR3 by* Porphyromonas gingivalis* for evading innate immune clearance might be a plausible hypothesis [41].

Signaling crosstalk between complement and Toll-like receptors (TLRs) normally serves to coordinate host immunity. However, the periodontal bacterium* Porphyromonas gingivalis* expresses C5 convertase-like enzymatic activity and adeptly exploits complement-TLR crosstalk to subvert host defenses and escape elimination. Intriguingly, this defective immune surveillance leads to the remodeling of the periodontal microbiota to a dysbiotic state that causes inflammatory periodontitis. Understanding the mechanisms by which* Porphyromonas gingivalis* modulates complement function to cause dysbiosis offers new targets for complement therapeutics [42].

### 2.7. *Porphyromonas gingivalis* and Neutrofils

Periodontopathogens, such as* Porphyromonas gingivalis*, have developed different strategies to perturb the structural and functional integrity of the gingival epithelium.* Porphyromonas gingivalis* adheres to, invades, and replicates within human epithelial cells [43]. Periodontitis represents an important model for neutrophil-mediated host tissue injury. Neutrophils, primed or stimulated by the presence or persistence of infection, express an elevated and excessive response. This, in turn, leads to tissue destruction mediated by neutrophil activity [44].

The triggering receptor expressed on myeloid cells 1 (TREM-1) is a cell surface receptor of the immunoglobulin superfamily with the capacity to amplify proinflammatory cytokine production and regulate apoptosis. Polymorphonuclear neutrophils (PMNs) are the first line of defense against infection and a major source of TREM-1. The differential regulation of TREM-1 by the* Porphyromonas gingivalis* gingipains may present a novel mechanism by which* Porphyromonas gingivalis* manipulates the host innate immune response helping to establish chronic periodontal inflammation [45].

### 2.8. *Porphyromonas gingivalis* and Signal Transduction Pathways

*Porphyromonas gingivalis* can modulate eukaryotic cell signal transduction pathways, directing its uptake by gingival epithelial cells. Within this privileged site,* Porphyromonas gingivalis* can replicate and impinge upon components of the innate host defense [46]. Elucidation of a possible association of genotypic profiles under either diseased or clinical healthy conditions is important for understanding the pathogenic characteristics of* Porphyromonas gingivalis*. Genotypic characterization of* Porphyromonas gingivalis* strains has revealed extensive heterogeneity in natural populations of this bacterium [47]. Regulation of hemin-responsive genes in* Porphyromonas gingivalis* may occur by a negative regulator as has been described in other pathogenic organisms [48].

Extracellular signaling during inflammation and chronic diseases involves molecules referred to as “Danger Signals,” including the small molecule adenosine. The primary gingival epithelial cells express a family of G-protein coupled receptors known as adenosine receptors, including the high-affinity receptors A1 and A2a and low-affinity receptors A2b and A3. A2a receptor antagonism and knockdown via RNA interference significantly reduced metabolically active intracellular* Porphyromonas gingivalis*. The gingival epithelial cells express functional A2a receptor and* Porphyromonas gingivalis *may use the A2a receptor coupled “Danger Signals” adenosine signaling as a means to establish successful persistence in the oral mucosa, possibly via downregulation of the proinflammatory response [49].

### 2.9. Recent Research on* Porphyromonas gingivalis*

Currently, researchers are trying to find exactly how the symbiosis among the bacterial populations within the mouth works, specifically between* Porphyromonas gingivalis* and* Aggregatibacter actinomycetemcomitans*, as they are the major causes of gingivitis. Their specific aims include seeing what components are shuttled between the bacteria as it has already been proven that neither bacterial species can survive without the other [50]. Also, more studies are focusing on the fimbriae, specifically finding ways to inhibit the minor fimbriae production as that would prevent the formation of a biofilm on the tooth [51]. Another major area of research is finding out how current methods to destroy these pathogens work such as ammonium nitrate. Even though solutions like these have been used for some time now, the exact mechanism of how they are cytotoxic is unknown [52].

Future research aiming at finding the reasons which cause the changes in the oral homeostasis to allow the growth of* Aggregatibacter actinomycetemcomitans* may give insight into novel prevention strategies for* Aggregatibacter actinomycetemcomitans*-associated periodontitis. Since* Porphyromonas gingivalis* infection is related to a typical periodontal ecopathology, the susceptibility to person-to-person transmission of* Porphyromonas gingivalis* pathogen may be controlled by periodontal treatment and emphasizing the significance of high standard oral hygiene [53].

Recent clinical and epidemiological studies have provided strong evidence for a significant association between rheumatoid arthritis and periodontitis. Moreover, our findings show that* Porphyromonas gingivalis*, the major etiologic factor in periodontitis, facilitates the development and progression of collagen induced arthritis [54].

*Porphyromonas gingivalis*, a major cause of chronic periodontitis, has also been implicated in the etiology of rheumatoid arthritis, specifically in anti-citrullinated protein/peptide antibody positive, based on its unique property among pathogens to express a citrullinating peptidylarginine deiminase enzyme [23].* Porphyromonas gingivalis* peptidyl-arginine deiminase has the ability to convert arginine residues in proteins to citrulline. Protein citrullination alters protein structure and function; hence, peptidyl-arginine deiminase may be involved in deregulation of the host's signaling network and immune evasion. Furthermore, accumulating evidence suggests a role for autoimmunity against citrullinated proteins in the development of rheumatoid arthritis [55].

The research of the effects of tea catechin epigallocatechin gallate on established biofilms and biofilm formation by* Porphyromonas gingivalis*, a major pathogen of periodontal disease, shows that catechin epigallocatechin gallate destroys established* Porphyromonas gingivalis* biofilms and inhibits biofilm formation [56].

The results from current research indicate that, when treated with LPS from* Porphyromonas gingivalis*, the EphB4 receptor on osteoblasts and the EphrinB2 ligand on osteoclasts may generate bidirectional antiosteoclastogenic and pro-osteoblastogenic signaling into respective cells and potentially facilitate the transition from bone resorption to bone formation. In bone remodeling, the Eph family is involved in regulating the process of osteoclast and osteoblast coordination in order to maintain bone homeostasis [57].

The leptomeningeal cells transduce inflammatory signals from peripheral macrophages to brain-resident microglia in response to* Porphyromonas gingivalis* lipopolysaccharide. The expression of Toll-like receptor 2 (TLR2), TLR4, TNF-*α*, and inducible NO synthase was mainly detected in the gingival macrophages of chronic periodontitis patients. The leptomeninges serve as an important route for transducing inflammatory signals from macrophages to microglia by secretion of proinflammatory mediators during chronic periodontitis [58].

## 3. Conclusion

*Porphyromonas gingivalis* is a major pathogen of severe adult periodontitis. Alveolar bone resorption is one of the most important factors in denture construction.* Porphyromonas gingivalis* causes alveolar bone resorption and morphologic measurements are the most frequent methods to identify bone resorption in periodontal studies [57]. The microbiota of the human oral mucosa consists of a myriad of bacterial species that normally exist in commensal harmony with the host.* Porphyromonas gingivalis,* an etiological agent in severe forms of periodontitis (a chronic inflammatory disease), is a prominent component of the oral microbiome and a successful colonizer of the oral epithelium [59].

*Porphyromonas gingivalis* lipopolysaccharide circulates systemically in over 50% of periodontal disease patients and is associated with increased matrix metalloproteinase. The low systemic lipopolysaccharide stimulates an inflammatory response in the left ventricle through metalloproteinase leading to a decrease in cardiac function [60]. In the outer membrane, vesicles of* Porphyromonas gingivalis* contain virulence factors such as lipopolysaccharide and gingipains. The sera from periodontitis patients had significantly stronger reactivity against an outer membrane vesicles-producing strain than the isogenic outer membrane vesicles-depleted strain. The gingipain-laden outer membrane vesicles may contribute to tissue destruction in periodontal diseases by serving as a vehicle for the antigens and active proteases [61].* Porphyromonas gingivalis* and* Aggregatibacter actinomycetemcomitans* can be transmitted from family member to family member and may cause periodontitis in the recipient individual [40].

Recent advances in the understanding of the pathomechanisms of* Porphyromonas gingivalis* may lead to the development of novel strategies for eradication of* Porphyromonas gingivalis* and treatment for periodontal diseases in the future.

The past decades of biomedical research have yielded massive evidence for the contribution of the microbiome in the development of a variety of chronic human diseases. There is emerging evidence that* Porphyromonas gingivalis*, a well-adapted opportunistic pathogen of the oral mucosa and prominent constituent of oral biofilms, best known for its involvement in periodontitis, may be an important mediator in the development of a number of multifactorial and seemingly unrelated chronic diseases, such as rheumatoid arthritis and orodigestive cancers. Orodigestive cancers represent a large proportion of the total malignancies worldwide and include cancers of the oral cavity, gastrointestinal tract, and pancreas. For prevention and/or enhanced prognosis of these diseases, a good understanding of the pathophysiological mechanisms and the interaction between* Porphyromonas gingivalis* and host is much needed [62].

*Porphyromonas gingivalis*, an etiological agent in severe forms of periodontitis, is a prominent component of the oral microbiome and a successful colonizer of the oral epithelium. This Gram-negative anaerobe can also exist within the host epithelium without the existence of overt disease.* Porphyromonas gingivalis* seems to be a highly adapted pathogen of the oral microbiome. The latest studies tend to focus on the research of its possible direct and indirect influence on certain systemic diseases such as atherosclerosis and reumatoid arthritis. Mutual relations between* Porphyromonas gingivalis* and other pathogens of the oral cavity are another subject of intensive studies.

The authors have used both latest as well as older reviews and studies to compile a comprehensive overview of this key periodontal pathogen.
